# Pharmacy Student Challenges and Strategies towards Initial COVID-19 Curriculum Changes

**DOI:** 10.3390/healthcare9101322

**Published:** 2021-10-04

**Authors:** Luyao Liu, Suzanne Caliph, Claire Simpson, Ruohern Zoe Khoo, Geenath Neviles, Sithira Muthumuni, Kayley M. Lyons

**Affiliations:** Faculty of Pharmacy and Pharmaceutical Sciences, Monash University, Parkville, VIC 3052, Australia; lliu0007@student.monash.edu (L.L.); Suzanne.Caliph@monash.edu (S.C.); csim0005@student.monash.edu (C.S.); rkho0002@student.monash.edu (R.Z.K.); gnev0001@student.monash.edu (G.N.); smut0004@student.monash.edu (S.M.)

**Keywords:** e-learning, healthcare education, clinical teaching

## Abstract

Due to COVID-19, tertiary institutions were forced to deliver knowledge virtually, which proposed challenges for both institutions and students. In this study, we aimed to characterize pharmacy students’ challenges and strategies during COVID-19 curriculum changes, therefore developing a comprehensive understanding of students’ learning, wellbeing, and resilience in the ever-changing situation. Data were collected from student written reflections across four year levels at one school of pharmacy from March–May 2020. In addition, data were collected from written responses of second-year pharmacy students responding to prompted questions. The data were qualitatively analyzed inductively by five coders using NVivo 12. For each piece of data, two coders independently coded the data, calculated the inter-rater agreement, and resolved discrepancies. The most coded challenges were ‘negative emotional response’ and ‘communication barrier during virtual learning’. The most coded strategies were ‘using new technology’ and ‘time management’. This study allows researchers and education institutions to gain an overview of pharmacy students’ experiences during COVID-19, therefore helping universities to provide students with necessary support and techniques on how to self-cope with COVID-19 as well as stressful events in the future.

## 1. Introduction

At the beginning of the COVID-19 global crisis, healthcare education institutions and their students underwent transformative change. Overnight, institutions cut placements, moved small-group learning to Zoom^®^, and delivered education virtually. The self-isolation and new virtual learning systems influenced students’ study and daily life, potentially resulting in negative impacts on some students’ well-being [1,2]. For example, a recent study in China reported that 24.9% of their medical student cohort experienced anxiety to some degree due to social distancing and a lack of interpersonal communication during the COVID-19 pandemic [3]. Students have reported several challenges and low satisfaction with engaging in virtual learning during COVID-19 [1,4]. In contrast, other authors have reported that health professions students have adapted well from the challenges and virtual learning has resulted in better attendance, engagement, and feedback as both teaching staff and students have created various ways to cope [5,6].

Due to these varied responses to online learning, scholars have been interested in exploring the factors that influence student satisfaction during COVID-19. Chen and colleagues have found that student satisfaction with online learning during COVID-19 was explained by the quality of the online platform, emotional changes, and communication with students [4]. Chiu proposes that self-determination theory can help explain student engagement during COVID-19 [7]. Self-determination theory encourages educators to support student autonomy, competence, and relatedness in online learning. Satisfaction of three basic needs will, therefore, improve students’ behavioural, emotional, and cognitive engagement [7,8].

The purpose of this study is to gain a comprehensive understanding of pharmacy student benefits, challenges and strategies during the first few months of the COVID-19 pandemic. By examining the fallout from the immediate transition from face-to-face learning to distance virtual learning, this study contributes to a better understanding healthcare students’ adaptability and well-being throughout the pandemic. There is limited research on what types of strategies students have implemented in response to COVID-19′s effect on their learning, well-being, and motivation. For example, we are extending the work by Chiu into higher education. Additionally, this study will add to the emerging literature of the types of benefits that COVID-19 has had on their learning and development as future health care professionals. Insights from this study will allow healthcare education institutions to identify necessary student supports for this current pandemic and any future interruptions. This study will also provide an insight for future researchers during this time of unprecedented stress on the students.

Research questions for this study as follows:

RQ1: What types of benefits did pharmacy students experience during COVID-19?

RQ2: What types of challenges did pharmacy students experience during COVID-19?

RQ3: What types of strategies did pharmacy students use during COVID-19?

## 2. Materials and Methods

### 2.1. Study Design

The study design was a qualitative case study at one university institution. Our aim was to characterize pharmacy students’ experience during the first few months of the COVID-19 pandemic. The study was conducted through the analysis of students’ written reflections relevant to COVID-19 curriculum changes. In total, 774 responses from March 2020 to May 2020 were analyzed using Nvivo 12. Our six coders were guided by the research questions to independently code students’ written responses, and a codebook was developed based on several theories.

Previous studies have utilized software log data, questionnaires, interviews, and more [3,4,6,7]. Though these data sources are beneficial for understanding our studied phenomenon, we add a new perspective by analyzing students’ naturally occurring reflections. We mined students’ written reflections for what they spontaneously say affected them during COVID-19. These responses may differ than if we asked students directly in a questionnaire or interview. Other researchers have also mined written reflections for insights on the effects of COVID-19 but these were conducted with a focus on educators [9] and graduate students [10]. Findyartini and colleagues also explored health professions students’ written reflections by looking at medical students in Indonesia [11]. Our study provides another case-study in this area to triangulate Finyartini and colleagues’ results. 

### 2.2. Participants and Study Context

The participants of this study included first, second, third, and fourth-year pharmacy students studying a Bachelor of Pharmacy (Honors) and/or Master of Pharmacy at Monash University in Melbourne, Australia. The program follows the Pharmaceutical Society of Australia’s National Competency Standards Framework for Pharmacists in Australia [12].

The data collection occurred from March to May in 2020 following emergency education changes due to COVID-19. A summary of changes we made are outlined in a recent commentary [13]. Overall, the curriculum followed a standardized flipped classroom model explained in recent investigations of our program [14,15]. For on-campus learning, each week the students complete the following activities: (1) one day of self-directed online learning, (2) a day of large-class interactive lectures, (3) two days of small group workshops (one facilitator for 30 students in teams of five), and (4) a final day of “close-the-loop” lectures to answer any question and solidify the material. Before the first few months of COVID-19, the students attended large lectures and small group workshops in person. After COVID-19, these activities were shifted to Zoom conferencing technology. Some of the lectures and workshops were shortened or replaced with interactive online modules [10]. Before COVID-19, students attended placements in hospitals and community pharmacies, taking more responsibility each year they progressed in the program. After the first few months of COVID-19, some placements were shifted to virtual placements and others granted students more responsibility to assist with the increased workload at the pharmacy.

At Monash University, all pharmacy students complete a skills coaching program. The data for this study were collected in the context of a skills coaching program. The purpose of the skills coaching program is for students to develop their professional skills by discussing and reflecting upon eight professional skills: problem-solving, oral communication, written communication, empathy, reflective practice, integrity, teamwork, and inquiry. Students are required to attend skill coach meetings every few weeks. Before every skill coach meeting, students write Personalized Learning Plans (PLPs) on an ePortfolio. After submitting to the ePortfolio, a skill coach (e.g., faculty member, practicing pharmacist) provides feedback on the students’ PLP.

### 2.3. Data Collection

Data were collected from two sources. The first data source was the students’ PLPs across all year levels. The second source of data was second-year students’ written answers to written prompts during a skill coach meeting discussing student challenges and strategies related to COVID-19.

For the PLPs, students have the liberty to talk about incidents that have affected their skills, and therefore COVID-19 was a frequent and naturally emerging topic. The PLPs were collected from March 2020 to May 2020, following the start of the COVID-19 outbreak. From all available PLPs, we only selected PLPs which contained the following words for this study: ‘corona’, ‘COVID-19′, ‘online’, ‘zoom’, ‘virtual’, ‘virus’, ‘lockdown’, ‘quarantine’, and ‘pandemic.’ Of the many written PLPs, there were a total of 879 PLPs that met this inclusion criteria (i.e., contained certain words). A further 67 PLPs were excluded by coders due to the lack of relevance with our study. Each PLP varied in length from 100 to 400 words.

In addition to the PLPs, we collected data from an in-class learning activity. During a skills coach meeting, second-year pharmacy students were asked prompts on a shared Google Doc^®^ to facilitate discussion in groups of 10-12 students and a skills coach. The prompts were “What impacts are you seeing from COVID19?” and “How are you supporting your well-being during this time?” We collected all available student responses—197 responses from the first prompt and 97 responses from the second prompt.

### 2.4. Ethical Considerations

The Monash University Human Ethics Low Risk Review Committee approved this study (Project ID 24477). At the Monash Faculty of Pharmacy and Pharmaceutical Sciences, the pharmacy students are enrolled in an education research registry. Each year all students were informed of education research projects and presented with the opportunity to opt out of having their student data used for education research. For this study, we removed any PLPs from students who have opted-out of the education research registry.

### 2.5. Data Analysis

To analyze the data, we used a directed content analysis approach according to our three research questions [16,17]. First, we developed a codebook based on relevant theoretical frameworks and previous literature on self-determination theory, motivational strategies, learning strategies, and time management [18,19,20,21]. Research question two was guided by self-determination theory. Research question three was guided by frameworks in motivational, learning, and time management strategies. We open-coded responses for research question one.

Self-determination theory. Self-determination theory (SDT) is an empirically based theory of human motivation, development and wellness [18]. In our research, SDT served as the primary theoretical framework for coding students’ challenges, investigating how the pandemic has been causing frustration on their three basic needs: competence, relatedness, and autonomy [22].

Motivational strategies. Motivation refers to a student’s willingness to engage and persist in a task. Motivational regulation strategies may be triggered when students experience problems with their ongoing level of motivation, learning, and performance [19]. The coding was developed based on seven motivation regulation strategies identified by Maenpaa including environment structuring, self-consequating, goal-oriented self-talk, efficacy management, emotion regulation, regulation of value, and interest enhancement [19].

Learning strategies. The codes for learning strategies were primarily developed from ten different types of learning strategies examined by Dunlosky and colleagues including elaborative interrogation, self-explanation, summarization, highlighting (or underlining), the keyword mnemonic, imagery use for text learning, rereading, practice testing, distributed practice, and interleaved practice [20]. Ten learning techniques were either summarized from literature indicating they might improve student performance or surveys that students reported using them most frequently. As we aimed to identify strategies in virtual learning, the techniques were intentionally selected as students should be able to practice them without assistance and supervision.

Time management. Self-learning regulation models mainly focus on three perspectives of managing time: planning, monitoring, and regulating [23]. Uzir et al. clearly demonstrated that students who actively and consistently use time management strategies were associated with higher academic performance, established the relationship between the use of time management strategies and learning performance in blended learning of a health science course [21].

Six coders (LL, CS, RK, GN, SM, and KL) applied the codebook to the data using Nvivo12^®^. First, the six coders underwent a calibration phase by coding the data together. During the calibration phase, the coders added emerging codes and built group agreement for accurately coding the data. Then, five coders coded all of the second-year pharmacy student written responses together. After the calibration phase, the PLP data set (879 PLPs) were divided into teams of two coders. For each PLP, two coders independently coded the PLP. During this process, pairs of coders met to calculate inter-coder reliability and resolve their coding disagreements. The overall team of six coders met frequently to discuss changes to the codebook and KL frequently audited all of the team’s coding output.

After coding 480 out of the 879 PLPs, the team decided it had reached a saturation point and ceased coding. The saturation point was defined as the time when coding did not produce any new codes and the data coded under each code was often redundant [24]. After coding had ceased, the team calculated a final inter-rater agreement rate. Following coding, we identified themes and categories according to the established research questions. Frequent and salient themes were reported.

## 3. Results

The simple inter-rater agreement rate for all codes was 80.3%. The final codebook is included in Table 1. The results are organized according to research question.

### 3.1. What Types of Benefits Did Pharmacy Students Experience during COVID-19?

Although to a lesser extent than the challenges, many students discussed how COVID-19 had resulted in positive impacts on their studies and lives. Although we did not originally plan to investigate the benefits of COVID-19, it became an important and emerging research question. The benefits pharmacy students gained during COVID-19 depended on their experiences. In general, students on placement during COVID-19 shared positive practice experiences, whereas students completing remote learning benefited from ‘more time for themselves, family, friends, and Uni’ and ‘less travel commuting’.

Overall, of the benefit codes, having positive placement experiences was the most frequently coded theme (n = 27 instances). For some students, their placements during COVID-19 were the most experiential of their learning experiences. For example, one student said, “in my time at [hospital] amidst the COVID-19 pandemic, I had my most productive & growth-driven placement that has fundamentally changed how I approach my patients even as a student pharmacist.” Students discussed various reasons for why their placements were valuable during this time. Several students said they were “contributing”, “helping the pharmacy” and “a part of the team”. Students also thought their placements were interesting as they witnessed firsthand how pharmacies handled the fallout from pandemic.

The second most frequently coded theme was less travel commuting (n = 14). Transitioning to virtual learning meant some students “can manage [their] study at [their] own time” and “catch up with uni work” because they “spend less time travelling to uni”. Some students appreciated staying at home to enjoy more family time (n = 14), more friend time (n = 4) and more self-time (n = 7).

### 3.2. What Types of Challenges Did Pharmacy Students Experience during COVID-19? 

Similar to the benefits, the challenges of COVID-19 depended on the setting. We identified three main themes under challenges using SDT: Autonomy frustration, relatedness frustration, competence frustration. However, challenges also varied whether students were participating in virtual learning, placements, part-time pharmacy job, and a group inquiry (i.e., research) project (See Table 2).

Autonomy frustration refers to the feeling of no choice when students are carrying out an activity [22]. For example, students discussed in their reflections about how they were negatively impacted by the travel ban. The travel restriction (n = 12) has particularly impacted on the students coming from/planning to travel to the regions on the travel ban list, resulting in ending their trips early or cancelling their future trips, especially international students.

Competence frustration refers to feeling incapable of carrying out an activity [22]. For example, some students were struggling with managing their time (n= 22) and keeping up to date with their study (n = 22). Without the accountability of physically attending lectures and workshops, some of them “found [themselves] tend to procrastinate the work and the work [kept] piling up day by day”, and sometimes even worse, “[they] tend to forget that [they] actually still have classes and accidentally plan [ned] something on the time slot when [they] should be attending classes”. Students who were unable to manage their time generally felt “rushed”, “stagnant”, “overwhelmed” and eventually “lost in [their] study”.

Relatedness frustration refers to a lack of sense of belonging and connection to others [22]. As one of most coded themes, social isolation was coded 49 times (n = 49). Students were upset with entertainment restriction (n = 5) and travel restriction (n = 12). As one student illustrated, “while I love my alone time, finding anything to look forward to is a challenge”. Unfortunately, a few students reported observing or personally experiencing racism (n = 6) during their part-time job in pharmacies. Students described “unfair”, “feel attacked”, and “upset” about the distressing incidents.

Emotional responses (n = 147). As one of the most coded themes, emotional responses refer to any negative emotions such as stress and anxiety. The emotional responses were related to curriculum changes, social isolation, placement and working environment changes and the risk of getting infected during the pandemic. Some students “lost [a] sense of routine” in virtual learning; some students “[felt] terrible about staying at home only”; some students were “overwhelmed to attend the placement during a pandemic” and some students were worried about serving an infected patient.

For those students participating in virtual learning, the most frequently coded theme was communication barriers (n = 68), followed by difficulty adapting to the virtual environment (n = 43). For some students, communication with their peers via virtual meetings was much more difficult than face-to-face communication. One of the barriers was the lack of body language (n = 11). The students said they “can’t see visual cues” and it was “hard to make eye contact”, making it harder to communicate virtually. Lack of engagement (n = 26), motivation (n = 26), and teammate contribution (n = 19) have worsened students’ virtual learning experiences, especially with group assignments. Students complained that they “[feel] oddly disconnected during group meetings because people tend to stay silent” and “[they] don’t feel like [they] are learning or contributing”.

For the students that were undertaking a placement at the time, many challenges were posed against them. This mostly included fourth-year pharmacy students who were assigned two 4-week placement blocks. Many other students also reflected on their part-time job in the pharmacy. The most frequently coded challenge was a busy pharmacy (n = 63) and angry and difficult customers (n = 61). An example of a busy pharmacy response from a student includes “this was the most busiest and strangest experience for me in pharmacy”. In addition, they were required to supply limited stock to patients who tried to stockpile medications (n = 45) due to stock shortage (n = 32). Many students witnessed “the rush of medication hoarding”, which raised their concern for continuing care as “regular elderly patients [unable] to get their medications due to low stock”. In addition, some placement students had very limited placement exposure (n = 37) due to COVID-19.

### 3.3. What Types of Strategies Did Pharmacy Students Use during COVID-19?

We identified four main categories under strategies: mental and physical wellbeing, learning strategies, time management, and motivational strategies (See Table 3).

Physical and mental well-being. In terms of mental and physical wellbeing, COVID-19 preventative measures was the most coded theme (n = 84). Students emphasize that they have been practicing preventative and one student reflected, “As a pharmacist in training, this is an important lesson to be particularly vigilant and be role models for other students, friends and families”. Following by COVID-19 preventative measures, virtual social activities (n = 55) and leisure activities (n = 51) were the next two most coded themes. Some students have planned to “reach out family and friends” every day because they believe “stay connected” is very important during the ‘isolation period’. Meanwhile, doing leisure activities such as “pick[ing] a new hobby”, “baking”, and “watch[ing] TV shows” took students’ attention away from the current situation and their stress.

Learning strategies. Not every one of Dunlosky’s learning strategies was coded from students’ reflection [20]. However, students have developed their own learning strategies in response to virtual learning. The most frequently coded strategy from Dunlosky was distributed practice (n = 25); implementing a schedule to spare their learning activities evenly over time. For example, one student commented, “I just have to get into a routine of studying during study time, while giving myself breaks so that I don’t wear out too soon.” Most students have also employed their own learning strategies to assist with their continued learning. Most coded strategy was the use of new technology (n = 169) (e.g., Google Calendar) to adapt themselves to the virtual learning environment, especially for group communication and collaboration.

Time management. Most of the students acknowledged the importance of managing their time appropriately, especially in independent learning. Planning time ahead (n = 107) was one of the most coded themes in the codebook. Students implemented different tactics of planning their time ahead of study to help them keep on track. However, only one student mentioned evaluating use of time after the task was completed (n = 1).

Motivational strategies. Out of motivational strategies, the most frequently coded theme was goal-oriented self-talk (n = 21), which refers to the process that students think about various reasons for persisting or completing a task. For example, one student said, “If I ever get into a low head space with little motivation, I will remember my goal of wanting to help people which will put me back on track.” Students also reported that they were motivated by peer support. Students appreciated the support from friends and team members, motivating them to “keep [themselves] accountable in studying”. On the other hand, some students have also employed the same strategy to encourage their team members by “sharing how well [they] have worked so far and appreciating each member’s contribution to the team.

## 4. Discussion

Our study characterized pharmacy students’ benefits, challenges and strategies during the COVID-19 pandemic. Although previous literature has focused on students’ mental and learning challenges during pandemic [1,2,3], our research results provide an overview of both positive and negative pharmacy students’ experiences in specific contexts (e.g., placement, virtual learning). Overall, the transition to virtual learning was welcomed by some students and challenging for others. In placements, students experienced novel situations. However, students working and learning at community pharmacies experienced work demands, racism and difficult patient encounters. Nonetheless, students have implemented various strategies in order to overcome these difficulties and adapt to the new learning environment.

Similar to previous research [1], we found that the emergency implementation of virtual learning created new dynamics and posed new challenges for the students. In terms of self-determination theory, many students experienced competence frustration due to the abrupt transition to virtual learning. The competence frustration may have been attributed to a lack of structure [25]. In this case, students no longer have the structure of the campus, going to the library, starting their day, and hallway conversations with peers. As a potential result, many students described their struggles with personal accountability, procrastination, and time management. Deci and Ryan also outlined that the frustration of basic psychological needs can thwart the autonomous motivation therefore negatively affect the academic performance and achievement [18,26,27]. In addition to motivational strategies, the educators might consider implementing strategies to support students’ basic psychological needs in virtual learning. For example, educators might consider including students in decision-making of emergency changes to encourage student empowerment.

In particular, students cited poor-quality teamwork in virtual settings due to a lack of effective communication, motivation, and active engagement of all team members. The overwhelming descriptions of poor-quality teamwork is especially important considering that these students will work in healthcare in their career. These poor-quality teamwork experiences may discourage students from working collaboratively in the future. Therefore, educators may need to modify their approach. Just as multiplayer games and sport teams, group assignments requiring a strong team spirit can enhance students’ relatedness satisfaction [8]. For example, educators may design the virtual group assignments to be more competitive to ensure effective engagement and contribution. Additionally, it might be necessary to consider liaising with students regarding expectations of active engagement in a virtual learning environment, such as switching on the webcam during lessons. Although previous researchers have shown that the characteristics of educators do not significantly influence students’ learning outcomes in computer supported collaborative learning, they are still pivotal in relieving students’ anxiety therefore improving students’ academic performance [28].

Our research findings suggest that the majority of pharmacy students did not tend to employ learning strategies that were summarized from previous literature [20]. In a study conducted by Wolters et al., focusing on motivational regulation strategies, Wolters also found out that “students do not use all the types of motivational strategies equally” [29]. Although it is inspiring to see many students report developing their own strategies to overcome challenges and adapt to the new learning environment, this may reveal the need to educate the students on evidence-based approaches and introduce more advanced learning and motivational strategies. Due to the nature of the data source, the effectiveness of these self-developed strategies was not assessed. Therefore, future research that evaluates these strategies in the context of pandemic may be required.

In contrast, some students perceived some aspects of virtual learning as advantageous due to ease of accessibility and flexibility. Universities should apply what they learned from the emergency delivery of virtual learning to their future offerings. For example, our results suggest that students may prefer a combination of virtual learning on-campus learning. However, our research did not explore the correlation between virtual learning and academic learning outcomes and future research should continue to explore the affordance, effectiveness and barriers of blended learning models in the new post-COVID-19 reality. Specifically, future researchers could explore the impacts on educators, resources, and other types of students.

In addition to the findings that are corresponding to previous literature, our results provide an insight into the exclusive challenges faced by pharmacy students who went on placement or were participating in part-time jobs at local pharmacies. During the COVID-19 pandemic, some pharmacy students encountered limited placement exposure due to COVID-19 regulations in hospitals. On the other hand, students in community pharmacies felt that they were expected to handle excessive and unreasonable demands from customers in community pharmacy. In these cases, students may require individualized support from the faculty, in response to the rapidly changing placement and working environment. For example, educators might consider assigning additional assessment to students with limited exposure or providing additional training to students regarding how to handle these tough situations [13]. Our research is only limited to students’ experiences, and future research might explore faculties’ and preceptors’ perspectives.

However, some students reported only the benefits of COVID-19 on their placement experience. These future pharmacists were able to contribute more in their placements than if COVID-19 had never occurred. This increased participation in practice may then contribute to students’ professional identity formation [30] and sense of belonging [31]. When students participate in real-world communities of practice, they learn theories and skills in ways that are more readily applied in future careers [32].

Pharmacy students in our study reported heightened negative feelings and emotions during the pandemic, similar to a recent study by Zhai and colleagues involving medical students [2]. Factors that contributed to the negative emotions might be related to unfamiliar learning environments and placement experience and universal uncertainty during the pandemic. Many students have employed different tactics to support their mental health throughout the hard time. However, our results show that only a handful of students have self-reported utilizing the counselling service available at the University. Accordingly, it may be seen as an opportunity for the educators to encourage the pharmacy students to take advantage of the supporting services provided by the tertiary institutions. In addition to current existing services, educators may consider developing new programs to support students’ mental health during COVID-19 pandemic.

Our research finding suggests that autonomy and relatedness frustrations were related to the international students and students who live far away from their families. It is well established that the fulfilment of each basic psychological need is determinative with regard to an individual’s daily well-being [8]. While a large number of students reported experiencing negative emotion towards the pandemic as per our research results, specific plans may be tailored for individuals, specifically for international students and those who live far away from home. For example, the educators may consider scheduling group meetings for those students and a sense of belonging can be enhanced by sharing their experience with people in similar situations [33].

Our study was not without limitations. A limitation of our study was that we were unable to identify the year level of each student from the data source. To better aid student development, future researchers could evaluate the impacts and needs for each stage of student development. Although this research provides an in-depth view of one pharmacy cohort, any national or international policies should also account for related research in other cohorts, professions, institutions, and countries. Further, the data source used in this study is limited. Although analyzing student reflections reveals topics of central importance to students, some students may have written their reflections as an academic exercise, thereby “going through the motions” while writing their PLPs. In addition, it is possible that some students may be more challenged to express themselves in writing than in an interview or focus group study, whereas others may be more apt to express themselves through written reflections. Furthermore, the research provides first-hand information on pharmacy students’ experience in the early stage of the pandemic. However, due to the time constraint, we were not able to follow up any possible changes over time. The future research might compare students’ experiences in the early stage and the experiences in the later stage to better understand how students adapt to the COVID-19 pandemic. In addition, since the data were collected for other purposes (i.e., teaching and learning), there was a missed opportunity to comprehensively address all of the theoretical frameworks. For example, we were unable to explore social support factors for self-determination theory.

## 5. Conclusions

This study provides a high-level overview of pharmacy students’ benefits, challenges and strategies during the initial months of the COVID-19 pandemic. Our result demonstrated that the most coded challenges were “negative emotional response” and “communication barrier during virtual learning”. The most coded strategies were “using new technology” and “time management”. These research findings may help researchers and educators better understand students’ well-being and adaptability during the COVID-19 pandemic. Future researchers should investigate the long-term effects of COVID-19 on health professions students. Overall, tertiary institutions and educators may use the research findings to provide support that better suits the pharmacy students’ needs in the ongoing pandemic and any future emergency events.

## Figures and Tables

**Table 1:** The types of benefits that pharmacy students experienced during initial COVID-19 changes.

Theme	Number of References (*n* = )	Student Quotes
Having satisfying placement experiences	*n* = 27	“As such, I was able to see the positive impact of a level-headed and knowledgeable health professional on an anxious patient” “This position has given me great exposure, learning to dispense in a hospital, understand how our healthcare system is adapting to flatten the curve and even given me a chance to work on a COVID ward”
Less travel commuting	*n* = 14	“I can manage my study at my own time” “I can catch up with Uni work because I spent less time travelling to Uni”
More family time	*n* = 14	“Getting to spend more time at home with family members” “Calling my family and friends back in Malaysia more often”
Feeling valued and helpful during the pandemic	*n* = 13	“I felt a sense of responsibility in being able to juggle assisting and being of value to the pharmacy team during this demanding time”

**Table 2:** The types of challenges that pharmacy students experienced during initial COVID-19 changes.

Theme	Number of References (*n* = )	Student Quotes
Challenges of working in community pharmacy (part-time job and placement)	*n* = 256	“Felt overwhelmed by extra workload” “The pharmacy I work at has become 10 times busier than usual to the point where I have to work overtime unpaid, and get feverish from trying to remain calm with patients whilst maintaining empathy for our patients” “Some customers are frustrated, unwilling to wait and abusive to staff members”
Emotional responses	*n* = 147	“It has caused a lot of anxiety and frustration” “I have also continued to maintain professionalism in my work place as well as volunteer at the COVID-19 screening but it can be very mentally draining” “I found it quite disheartening and confronting, as this virus shows no rate of slowing down and its only getting worse and worse”
Communication barrier in virtual learning	*n* = 68	“Due to workshops occurring online, it was much more difficult for students to openly communicate with one another.” “No one really wants to talk unless asked a question”
Social isolation	*n* = 49	“Being by myself has been a struggle because I can’t meet my friends on weekends” “This has been hard for me as I live alone so I feel like I have lost all of my human interaction”
Difficulty adapting to online environment	*n* = 43	“I was completely lost with the new virtual learning environment” “I felt very uncomfortable and distracted to study with my camera on me as it creates a feeling of under surveillance”
Technical issues during virtual learning	*n* = 26	“I saw a message on top of my screen, stating that my “connection is lost” and this was when I freaked out even more.”

**Table 3:** The types of strategies that pharmacy students used during initial COVID-19 changes.

Theme	Number of References (*n* = )	Student Quotes
Mental and physical well-being	*n* = 362	“I feel like that it is a very normal thought to have after talking to all my friends and opening up to them about my feelings” “Furthermore, practices of mindfulness that develop skills of resilience to help students to alleviate stress and objectively review the situation.” “I will also try to reach out to a friend everyday and see how they are doing and offer support and relief the best I can”
Learning strategies	*n* = 285	“Eventually, I came up with the idea to work on the project via zoom, so we worked together, tapping in and out of the document and zoom when we needed to. This was far more efficient than working separately and waiting for the rest of the group members to see changes to the document and were able to complete and submit the report a day early” “We decided to make a group chat on messenger, making it easier for all group members to keep updates and to communicate with each other.’
Time management	*n* = 172	“Given the challenge of being at home and trying to keep on top of everything, I am going to make a timetable, that will separate my day into blocks, one block per subject.” “I will reflect on how effective my timetable was at the end of each day. I will also make modifications to my timetable every Sunday until the timetable is suitable with my current lifestyle”
Motivational strategies	*n* = 61	“By doing this, my stress levels will decrease since I am more organized and I can also avoid completing my assignments at the very last minute.” ‘It also acts as a way of keeping myself accountable in terms of studying, as we check up on each other’s progress daily, and it has also made me more motivated to study and stay on top of coursework as it is the only time we get to see each other.”
